# Effect of Intermittent Warming on the Quality and Lipid Metabolism of Blueberry (*Vaccinium corymbosum* L., cv. Duke) Fruit

**DOI:** 10.3389/fpls.2020.590928

**Published:** 2021-02-04

**Authors:** Hongyu Dai, Yajuan Wang, Shujuan Ji, Ximan Kong, Fan Zhang, Xin Zhou, Qian Zhou

**Affiliations:** ^1^College of Food, Shenyang Agricultural University, Shenyang, China; ^2^College of Plant Protection, Shenyang Agricultural University, Shenyang, China

**Keywords:** blueberry, storage, intermittent warming, lipid metabolism, fruit quality

## Abstract

The change of lipid metabolism is a key point of blueberry fruit after refrigeration. This study was conducted to evaluate the effects of intermittent warming (IW) of “DuKe” blueberry fruit on its shelf life at 20 ± 0.5°C following 30 days of refrigeration. IW-treated fruit showed higher contents of phosphatidylcholine, linoleic acid, and oleic acid but lower contents of phosphatidic acid and palmitic acid compared to controls. Protective effects on the cell membrane were also reflected as inhibition of the activity of phospholipase D and lipoxygenase. The blueberry fruit showed a lower decay and pitting incidence with higher firmness than control. Interestingly, IW increased C-repeat binding transcription factor gene expression, which can induce the expression of genes related to hypothermia tolerance in plant cells at low temperature. These results indicate that IW can prevent damage to the membrane lipids, which occurs by senescence at a low temperature of blueberry fruit.

## Introduction

Blueberry (*Vaccinium* spp.) is a kind of popular fruits because of high levels of nutrients (Wang S. Y. et al., 2018). The maintenance of quality is important to guarantee the economic success of fruits such as blueberry (Montecchiarini et al., 2019). However, the post harvest life of blueberry fruit is short. Therefore, it is necessary to improve the storage quality of fresh blueberry fruit using efficient strategies.

Intermittent warming (IW) is a convenient and environment-friendly strategy for delaying senescence and relieving chilling injury (CI) during low-temperature storage. For example, The IW treatment during cold storage can alleviate the peel browning of “Nanguo” pears (Wang J. W. et al., 2018). Fernández-Trujillo and Artés (1997) applied intermittent heating during storage of peach fruit, which effectively maintained the firmness and color of peach fruit. The role of IW treatment in reducing fruit chilling has been widely demonstrated, but few studies have evaluated the relationship between membrane lipid changes, cold stress, and IW treatment after low-temperature storage.

Membrane integrity is important for maintaining the quality of blueberry fruit during storage. The types and contents of unsaturated fatty acids and membrane lipids play important roles in lipid metabolism (Lyons, 1973; Marangoni et al., 1996). Lipids on the cell membrane are mainly phospholipids, glycolipids, and cholesterol. Phospholipid is the most important component, and its double-layer structure ensures the stability of various physiological reactions in the fruit. Phospholipids in plants are complex (Kong et al., 2018). However, phosphatidic acid (PA), phosphatidylcholine (PC), phosphatidylglycerol (PG), phosphatidylethanolamine (PE), phosphatidylserine (PS), and phosphatidylinositol (PI) are commonly present. Senescence and some abiotic stressors cause degradation of phospholipids on the cell membrane, damage to the structure and integrity of the cell membrane, and disruption of the compartments within the cell. Phospholipids not only function as a barrier between cells and the outside environment but also are extremely important signal molecules, which is critical for the conversion of fatty acids (Manoharan et al., 1990). Any shift in the compositions of phospholipid and fatty acid during storage may damage the structural integrity of the cell membrane system (Lin et al., 2016), during which the enzymes phospholipase D (PLD) and lipoxygenase (LOX) play a major role. In short, PLD initiates the hydrolysis of phospholipids, after which LOX catalyzes the oxygenation of polyunsaturated fatty acids. Attack by the peroxidation products results in loss of compartmentalization of the cell membrane system (Cai et al., 2015). In addition to changes in the saturation levels of fatty acids, the composition and content of phospholipids also change to a certain extent at cold temperatures (Tarazona et al., 2015).

Based on our previous studies on the changes of membrane lipid in blueberry fruits during cold storage, we found that after 30 days of refrigeration, the pitting incidence increased significantly, and the linoleic acid content decreased significantly on day 4 of shelf life at room temperature (Wang Y. J. et al., 2019). Therefore, this study aimed to analyze the changes of membrane lipid of blueberry fruits during shelf life after a 30-day refrigeration and we have chosen the data at day 4 of the shelf life as the main object to analyze the differences of fatty acid content and membrane lipid composition between the IW-treated fruit and the controls. In addition, for clarifying the changes of enzyme activities of PLD and LOX better, we have chosen two representative genes, *VcLOX1* and *VcPLD*β, based on our previous studies. We also found that some phenomena in fruit during low-temperature storage were similar to the results of CI in the previous study. Some transcription factors such as C-repeat binding transcription factors (CBFs) have a vital role in plants involved in response to low temperature (Gilmour et al., 2004; Zhao et al., 2016). It was reported that the ectopic expression of CBFs can increase the freezing tolerance of transgenic Arabidopsis (Yamaguchi-Shinozaki and Shinozaki, 2006). Some studies also showed that treatments such as salicylic acid and eugenol fumigation increased the tolerance of grape and eggplant fruit to cold stress by increasing the gene expression of CBFs (Aazami and Mahna, 2017; Huang et al., 2019). Based on the study of the role of CBF in plant cold resistance, we determined *VcCBF* gene expression levels in this study.

The purpose of this study was to investigate the effect of IW treatment on “DuKe” blueberry at room temperature after refrigeration. The membrane lipid composition and fatty acid content of IW-treated fruit on day 4 of the shelf life after a 30-day refrigeration (0 ± 0.5°C) were examined. The firmness, decay incidence, pitting incidence, electrolyte leakage, malondialdehyde (MDA) concentration, and proline concentration at days 0, 2, 4, 6, and 8 of refrigeration were evaluated to determine the influence of cell membrane damage. Moreover, the enzyme activities of LOX and PLD and gene expression of *VcLOX1*, *VcPLD*β, and *VcCBF* during the shelf life were evaluated.

## Materials and Methods

### Fruit Material and Treatments

Samples of blueberry fruit (*Vaccinium* spp. “DuKe”) without mechanical injury or disease were harvested at commercial maturity (firmness about 2.0–2.5 N, total soluble solids about 7–8%, purple surface area larger than red area) from Shenyang, Liaoning Province, China (41.8°N, 123.4°E).

After 10 h of pre-cooling at 0 ± 0.5°C, the fruit was randomly divided into two groups; each group was composed of 20 kg fruit and transplanted into plastic boxes. The fruit of each box contains 125 g. The control group was stored at 0 ± 0.5°C, while another group was treated with IW by exposing to 20 ± 0.5°C for 1 day every 10 days during refrigeration. Both groups were stored for 30 days and then placed for 8 days at room temperature (20 ± 0.5°C) and 80% RH.

Fruit from both groups were sampled for analysis at 0, 2, 4, 6, and 8 days during the shelf life. Three replicates of fruit from both groups at each sampling point were collected to determine the firmness, decay index, pitting incidence, and electrolyte leakage. The fresh samples were frozen in liquid nitrogen and stored at −80°C until use for the measurement of fatty acid, membrane lipid composition, MDA concentration, proline concentration, enzyme activity, and gene expression. Three independent replicates were conducted.

### Measurement of Decay Incidence

Decay incidence was expressed as percentage of the total number (Am) of blueberry fruits in each group. Three independent replicates were measured, and 100 fruit per replication were taken for analysis. Decay incidence was shown as follows: decay incidence = An/Am × 100%, where An is the number of blueberries with decay and Am is the total numbers.

### Measurement of Pitting Incidence

Three independent replicates were measured, and 100 fruit per replication were taken for analysis.

The pitting incidence of the sample was calculated according to the following equation: pitting incidence = An/Am × 100%, where An is the number of blueberries with pitting and Am is the total numbers.

### Measurement of Firmness

Fruit firmness of 10 fruit at each sampling point for each treatment was measured using a CT3 texture analyzer (Brookfield Engineering Laboratories, Inc., United States) with the TA39 rod probe. The penetration rate was 0.5 mm s^–1^, and the final penetration depth was 5 mm. The result was expressed in newton (N).

### Measurement of Electrolyte Leakage, MDA, and Proline Concentration

Electrolyte leakage measurements were performed following Zhu et al. (2009) using 15 disks with 1-mm thickness and 10-mm diameter from five fruits. Electrolyte leakage was expressed as relative conductivity (%).

Extraction of MDA content was according to Wang Y. J. et al. (2019). Blueberry tissue (1 g FW) from three blueberry fruit per replication has been taken for analysis. The MDA concentration (μmol g^–1^) was calculated according to the equation: [6.45 × (*A532* − *A600*) − 0.56 × *A450*] × 5.

The values of *A*532, *A600*, and *A*450 are the absorbance of the sample at 532, 600, and 450 nm.

Proline content was measured according to the method of Zhao et al. (2009) with slight modifications. Briefly, 2 g (FW) tissue from three blueberry fruit was suspended in 2 mL of 30 g L^–1^ sulfosalicylic acid and transferred into a test tube quickly. Subsequently, the tube was heated in a 100°C water bath for 10 min and cooled to room temperature and then centrifuged at 10,000 × *g* for 15 min. Then, 2 mL of the supernatant was mixed with a mixture of glacial acetic acid and ninhydrin. After that, the mixture was heated in a 100°C water bath for 30 min until red. After quickly cooling, 4 mL of toluene was added into the tube, which was shaken for 30 s and then allowed to stand still for a while. The absorbance was read at 520 nm using the UV 5100 spectrophotometer (METASH). The content was calculated according to the standard curve and expressed as μg g^–1^.

### Analysis of Membrane Lipid Composition

Membrane lipid extraction was performed according to Welti et al. (2002) with some modifications. The fruit of the harvest day, the control group, and the treatment group were used as the samples for determination (fresh sample, control sample, and IW-treated sample). Blueberry tissues cut from the same position from eight fruits were transferred into 3 mL of pre-heated dimethylcarbinol, containing 0.01% butylated hydroxytoluene (BHT), and incubated in a 75°C water bath for 15 min. Then, 1.5 mL of chloroform and 0.6 mL of ultrapure water were added into the tube successively and centrifuged for 1 h at 150 *g* min^–1^. The extract was transferred into the glass tube. The lipid extraction was repeated with 4 mL of CHCl_3_/MeOH (2:1, v/v) (containing 0.01% BHT) for 30 min at 150 *g* min^–1^ and then transferred again. The steps were repeated until the samples became discolored. After that, the extracted solutions were combined, followed by addition of 1 mL of 1 mol L^–1^ KCl and centrifugation at 500 × *g* for 5 min; the water phase was discarded. 2 mL of ultrapure water was added to the extract and centrifuged at 500 × *g* for 5 min, and the water phase was discarded. The solvent was evaporated under a stream of N_2_, stored at −80°C.

Membrane lipids were measured by the method as Xiao et al. (2010) described.

### Analysis of Fatty Acid Content

Blueberry tissue (5.0 g FW) from 15 fruits, whose enzymes were inactivated in the oven (100°C, 10 min) and cooled to room temperature (20°C), was used as samples for follow steps.

Fatty acids were extracted using the method of Zhang and Tian (2010) and assayed as described by Wang Y. J. et al. (2019).

The fatty acids were qualitatively analyzed using the NIST/WILEY MS Search 2.0 standard database.

### Analysis of Enzyme Activity

Blueberry tissue (5 g FW) from 15 fruits was fully ground to homogenate in 5 mL precooled phosphate buffer (0.1 mol L^–1^ Na_2_HPO_4_⋅12H_2_O, 0.1 mol L^–1^ NaH_2_PO_4_⋅2H_2_O) and centrifuged at 8,300 × *g* and 4°C for 20 min to collect the culture fluid to determine PLD activity according to the instructions provided in the Plant Phospholipase D ELISA Kit (Shangle, China). The PLD activity was expressed as μg L^–1^ FW.

Blueberry tissue (5 g FW) from 15 fruits was fully ground to homogenate in 5 mL precooled 0.01 mol L^–1^ phosphate buffer (pH 6.8) and centrifuged at 12,000 × *g* and 4°C for 20 min to collect the culture fluid to determine the LOX activity according to the instructions provided in the Plant Lipoxygenase ELISA Kit (Shangle, China). The LOX activity was expressed as μg L^–1^ FW.

### RNA Isolation, cDNA Synthesis, and Real-Time q-PCR Assays

Blueberry tissue (100 mg FW) from three fruits was ground into powder in liquid nitrogen and transferred into a 1.5-mL RNase-Free tube for total RNA extraction of each treatment and time point.

Total RNA extraction, synthesis of cDNA, and qRT-PCR were performed according to the kit instructions of OminiPlant RNA Kit (CWBIO, China), HiFiScript cDNA Synthesis Kit (CWBIO, China), and UItraSYBR Mixture (Low Rox) Kit (CWBIO, China), respectively. The gene-specific primers used for RT-PCR (*VcPLD*β, *VcLOX1*, and *VcCBF*) designed using Primer Premier 5.0 are shown in Table 1. The sample of blueberry fruit on the harvest day was used as the mock sample (not shown in this article) following the 2^–ΔΔCt^ method.

**TABLE 1:** Primers for real-time q-PCR analysis.

**Gene**	**Forward primer (5′–3′)**	**Reverse primer (5′–3′)**
*Actin*	5′-ACTACCATCCACT CTATCACCG-3′	5′-AACACCTTACCAACAGCCTTG-3′
*VcPLD*β	5′-TCAGCTTACGTCG TTATTCCTATGTG-3′	5′-ACGGTTGCCAAGACAGTAGAAGTTC-3′
*VcLOX1*	5′-GGATCACCATGAT GCGCTAA-3′	5′-ATGGCTTCAGTGTTCCGTCA-3′
*VcCBF*	5′-GCCTCTCGTCCTG CCGTATTAATG-3′	5′-TCCAGGTCAGGAATCAACAAGCAG-3′

### Statistical Analyses

All of the statistical analyses were conducted using SPSS 20.0 (SPSS Inc., Chicago, IL, United States). Data were analyzed by one-way analysis of variance (ANOVA). Means were analyzed using Duncan’s multiple-range tests. All of the differences were considered significant at *p* < 0.05.

## Results

### Changes in Decay Incidence, Pitting Incidence, and Fruit Firmness During the Shelf Life of Refrigerated Blueberry Fruit at Room Temperature

**FIGURE 1:**
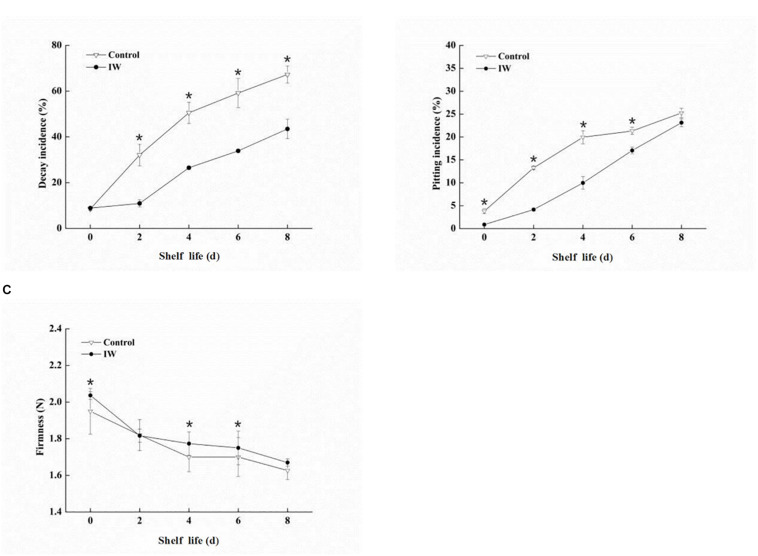
Effect of intermittent warming on decay incidence **(A)**, pitting incidence **(B)**, and firmness **(C)** of blueberry fruit during shelf life at room temperature after a 30-day refrigeration. Mean ± SE of three replicate experiments are shown. Asterisks indicate significant differences between control and IW-treated fruit (*p < 0.05).

From day 0 to day 8 at room temperature after a 30-day refrigeration, the decay incidence (%) of the blueberry fruit increased quickly in both two groups (Figure 1A). At 2, 4, 6, and 8 days of the shelf life, there were significant differences between IW-treated and control fruit (*p* < 0.05). The decay incidence was more than 67% in control fruit the last day; in contrast, it was only 43% in IW-treated fruit, indicating that the treatment reduced fruit loss by more than 20%.

There was a sharp increase in pitting incidence during the shelf life at room temperature, and the incidence of pitting in control fruit was higher than that in IW-treated fruit (Figure 1B). IW treatment significantly alleviated the occurrence of pitting incidence until day 6 (*p* < 0.05). In particular, the incidence of pitting in IW-treated fruit was detected to be 11 and 10% lower than that of control fruit on days 2 and 4, respectively.

During the whole shelf life at room temperature, the firmness exhibited a normal softening pattern in both two groups (Figure 1C). After a 30-day refrigeration, the firmness of the IW-treated fruit was significantly higher than that of the control at 0, 4, and 6 days (*p* < 0.05).

### Changes in Electrolyte Leakage, MDA Concentration, and Proline Concentration During the Shelf Life of Refrigerated Blueberry Fruit at Room Temperature

**FIGURE 2:**
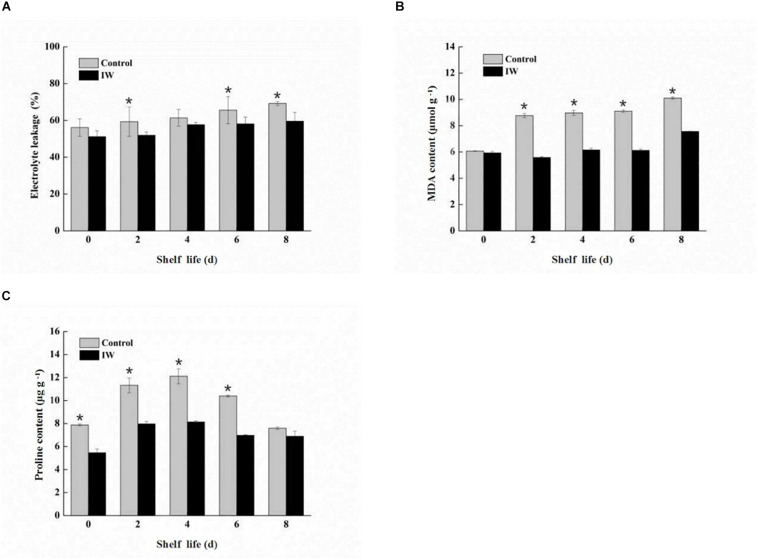
Effect of intermittent warming on electrolyte leakage **(A)**, MDA content **(B)**, and proline content **(C)** of blueberry fruit during shelf life at room temperature after 30 days refrigeration. Mean ± SE of three replicate experiments are shown. Asterisks indicate significant differences between control and IW-treated fruit (*p < 0.05).

The electrolyte leakage of both two groups increased steadily during the shelf life after the 30-day refrigeration (Figure 2A). Compared with the control group, the electrolyte leakage of IW-treated fruit was maintained at a lower level; especially at 2, 6, and 8 days, the differences between the two groups were significant (*p* < 0.05).

At the beginning of the shelf life, there were no significant differences between the fruit of two groups (Figure 2B), and both of two increased steadily. However, the MDA concentrations in IW-treated fruit were significantly lower than those in control fruit from 2 to 8 days (*p* < 0.05).

During the entire shelf life, the proline concentrations of IW-treated fruit were increased in the first 4 days of shelf life and then decreased, and the peak appeared in 4 days. The level of control fruit showed a similar trend to IW-treated fruit. Proline concentrations of IW treatment were lower than control, and the differences between the two groups were significantly different at 0, 2, 4, and 6 days (*p* < 0.05) (Figure 2C).

Compared with the control fruit, the accumulation of membrane lipid peroxidation markers in the treatment group was significantly reduced (*p* < 0.05), indicating that the stability and integrity of the cell membrane were well protected.

### Effects on Membrane Lipid Content on Day 4 of the Shelf Life at Room Temperature of Refrigerated Blueberry Fruit

We identified two categories of galactolipids [monogalactosyldiacylglycerol (MGDG) and digalactosyldiacylglycerol (DGDG)], six categories of phospholipids [PA, PC, PE, PG, PI, and PS], and three categories of lyso-phosphatides [lyso-phosphatidylcholine (LPC), lyso-phosphatidylethanolamine (LPE), and lyso-phosphatidylglycerol (LPG)].

**FIGURE 3:**
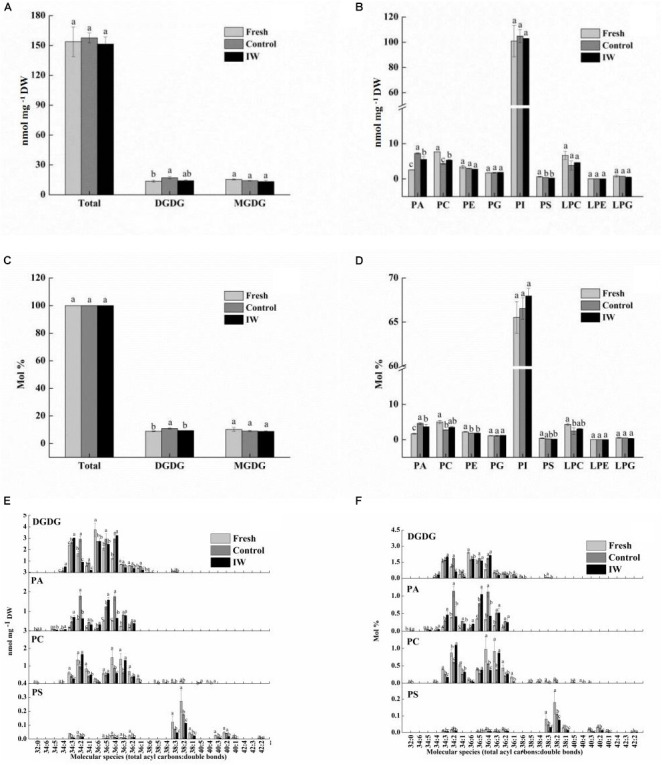
Effect of intermittent warming on membrane lipid content of blueberry fruit at the day 4 during shelf life at room temperature after 30-day refrigeration. **(A)** Total lipid and glycolipid composition changes during shelf life, **(B)** phospholipid composition changes during shelf life, **(C)** total lipid and glycolipid proportion changes during shelf life, **(D)** phospholipid proportion changes during shelf life, **(E)** main glycolipid and phospholipid molecular species composition changes during shelf life, and **(F)** main glycolipid and phospholipid molecular species proportion changes during shelf life. Mean ± SE of three replicate experiments are shown. The letters a, b, and c show significant differences according to the independent samples *t*-test (*p* < 0.05) at each time point.

The total lipid contents were 153.8, 157.7, and 151.5 nmol mg^–1^ DW in fresh, control, and IW-treated samples, respectively (Figure 3A). The contents of DGDG were significantly higher in control fruit than in fresh fruit (*p* < 0.05), whereas the contents in IW-treated fruit were not. Compared to fresh fruit, the contents of PS and PC in control fruit and IW-treated were significantly decreased (*p* < 0.05) (Figure 3B); the contents of PC in IW-treated fruit were higher than in controls. In contrast, the contents of PA were increased after refrigeration, and they were significantly lower in IW-treated fruit (*p* < 0.05). The relative change is shown in Figures 3C,D.

The differences in the contents of PA came from the increases in 34:2 and 36:4 PA. Some of the PC and PS contents decreased significantly, such as 34:2 and 36:3 PC and 36:4 and 38:2 PS (Figures 3E,F). Under low-temperature stress, the structure of non-bilayer lipid MGDG is easy to transform into DGDG with a relatively stable structure and the increase of DGDG content indicated that the fruit was subjected to low-temperature stress. In this study, there are five main molecular species of DGDG, including 34:2, 34:3, 36:4, 36:5, and 36:6. Higher levels of molecular species of 34: 3-, 36: 4-, and 36:5-DGDG occurred along with a lower level of 36:6 DGDG in control and IW-treated fruit than in fresh fruit. However, the main components affecting the difference of DGDG content between control and IW-treated fruit may be 34:2 DGDG (Figures 3E,F).

### Effects on Fatty Acid Content and the Index of Unsaturated Fatty Acid at Day 4 During Shelf Life of Refrigerated Blueberry Fruit at Room Temperature

Five major fatty acids were detected, namely, palmitic acid and stearic acid as saturated fatty acids, oleic acid as monounsaturated fatty acid, and linoleic acid and linolenic acid as polyunsaturated fatty acids.

**FIGURE 4:**
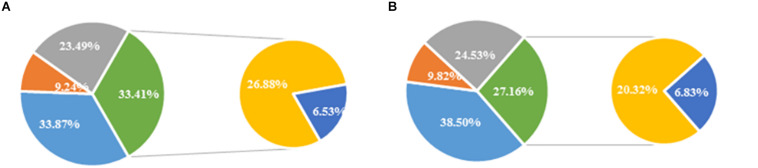
The fatty acid content of blueberry fruit at the day 4 of shelf life at room temperature after 30 days refrigeration in control **(A)** and intermittent warming **(B)**. Mean ± SE of three replicate experiments are shown.

At day 4 of the shelf life following a 30-day refrigeration, the content of stearic acid and linolenic acid in IW-treated fruit was higher than controls, although the difference was not significant (Figure 4). The content of palmitic acid was significantly lower (*p* < 0.05). At the same time, the content of oleic acid and linoleic acid was significantly higher than controls (*p* < 0.05). Overall, the relative content of saturated fatty acids in the control blueberry fruit was 26.88%, which was more than 6% higher than that in the IW-treated fruit (20.32%). As shown in Table 2, both the indexes of unsaturated fatty acid (IUFA) and unsaturated fatty acid/saturated fatty acid (UFA/FA) in control fruit were lower than those in IW-treated fruit (*p* < 0.05).

**TABLE 2:** Effect of intermittent warming on index of unsaturated fatty acid of blueberry fruit at the day 4 of shelf life at room temperature after 30 days refrigeration.

	**IUFA**	**UFA/FA**
Control	122.797	1.993
IW	131.716*	2.682*

Values are meaning ± SD. Asterisks indicate significant differences between control and IW-treated fruit (*p < 0.05, **p < 0.01).

### Changes in the Enzyme Activity and Expression Level of Genes of Refrigerated Blueberry Fruit at Room Temperature

**FIGURE 5:**
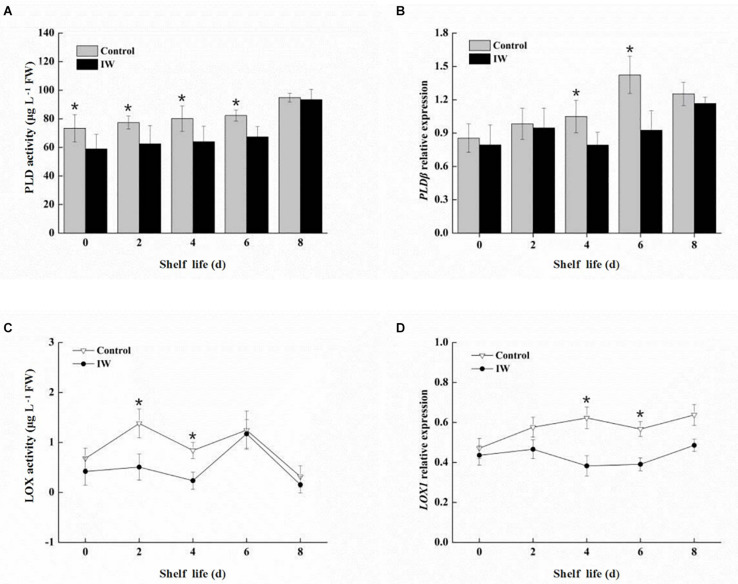
Effect of intermittent warming on PLD activity **(A)**, gene expression of *VcPLD*β **(B)**, LOX activity **(C)**, and gene expression of *VcLOX1* **(D)** in blueberry fruit at room temperature after a 30-day refrigeration. Mean ± SE of three replicate experiments are shown. Asterisks indicate significant differences between control and IW-treated fruit (*p < 0.05).

The activity of PLD was increased in both two groups after a 30-day refrigeration, and its levels were lower in IW-treated fruit than in the control fruit (Figure 5A). Significant differences in activity were observed (*p* < 0.05) during the first 6 days post refrigeration. IW treatment suppressed the increase in PLD activity during the period post refrigeration. The expression levels of *VcPLD*β showed similar trends in the two groups, and the levels were significantly lower in IW-treated fruit than in the control fruit (*p* < 0.05) (Figure 5B). However, in control fruit, levels of *VcPLD*β were peaked at day 6, while in IW-treated fruit, they peaked at day 8. Therefore, IW treatment delayed the peak of the expression of *VcPLD*β.

The LOX activity in IW-treated fruit showed the same trend as in control fruit, and the levels in these fruit were similar at day 6 and day 8 after a 30-day refrigeration (Figure 5C). The level in IW-treated fruit during the first 4 days was lower than in controls, and the difference in levels was significant (*p* < 0.05). Compared to the control, IW treatment could decrease the level of LOX activity and delay its peak activity. There was no significant change in the expression levels of *VcLOX1* in IW-treated fruit post refrigeration (Figure 5D). At the same time, the expression level in the control increased slowly and was higher than that in IW-treated fruit; differences in expression levels were significant at day 4 and day 6 (*p* < 0.05).

### Changes in the Expression Levels of *VcCBF* Post Refrigeration of Blueberry Fruit at Room Temperature

**FIGURE 6:**
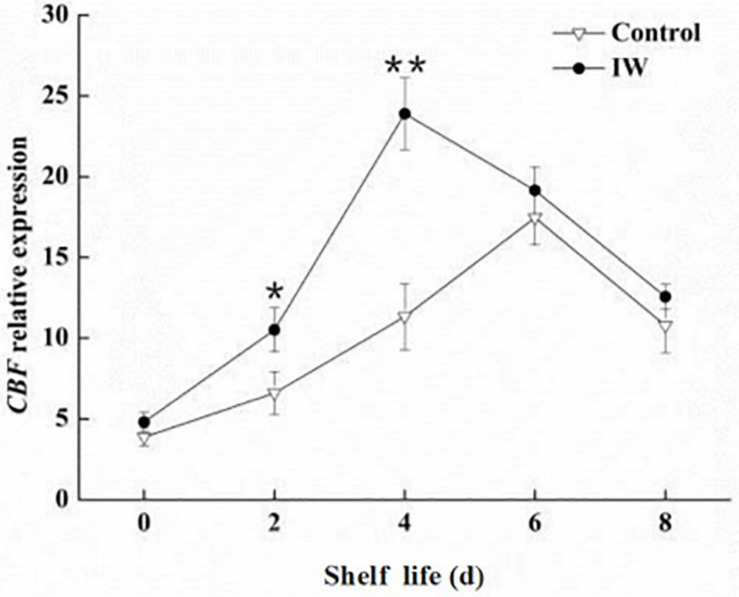
Effect of intermittent warming on the gene expression of *VcCBF* of blueberry fruit at room temperature after a 30-day refrigeration. Mean ± SE of three replicate experiments are shown. Asterisks indicate significant differences between control and IW-treated fruit (*p < 0.05, **p < 0.01).

The highest expression of *VcCBF* was observed at day 6 and at day 4 in control fruit and IW-treated fruit, respectively, after a 30-d refrigeration (Figure 6). The expression level in IW-treated fruit was significantly higher than that in controls at day 2, and the difference in the expression levels between the two groups was significant (*p* < 0.01).

## Discussion

Low-temperature storage is a common solution to the problem of off-season fruit supply (Sanchez-Bel et al., 2012). However, it is also associated with certain limitations; one of them is the damage of cell membranes. For example, pears and longan fruit are prone to browning after low-temperature storage (Wang J. W. et al., 2018; Zhang et al., 2019), whereas the main symptoms which showed damage due to low-temperature storage in peppers are surface pitting, seed darkening, and so on (Hardenburg et al., 1990). For blueberry, one of the main consequences of low-temperature damage is the pitting of the fruit. The development of pitting in blueberry fruit during low-temperature storage is rapid and damage to cellular membranes (Zhou et al., 2014). IW is widely used to maintain the quality of fruit during storage and has been demonstrated to be effective for fruits such as bell peppers (Kong et al., 2018), peach fruit (Xi et al., 2012), and apple fruit (Alwan and Watkins, 1999). In this study, IW treatment delayed the incidence of pitting in “DuKe” blueberry fruit after a 30-day low-temperature storage (Figure 1B) and maintained a higher degree of firmness and lower incidence of fruit decay (Figures 1A,C).

These functions appear to be mediated by effects on membrane components. As a key point of fruit ripening and senescence before the component content and enzyme activity changes of the cell wall, the change of membrane integrity and membrane penetrability deserve attention (Maalekuu et al., 2006; Meyer and Terry, 2010). Phospholipids, as the principle component of the plasma membrane, maintain cellular fluidity and permeability and play an important role in the maintenance of regular physiological metabolism. As a major phospholipid, PC undergoes hydrolysis and transfer under the action of PLD during fruit senescence, producing PA and another phospholipid, resulting in changes in cell membrane structure and function (Aktas et al., 2014). Intermittent temperature treatment effectively inhibits the degradation of PC, which means that the cell membrane structure can be better protected from damage. Phosphatidic acid can provide some substrates for lipid peroxidation by participating in a series of reactions (Jia and Li, 2015). The collapse of the membrane system caused by cell senescence causes a strong accumulation of PA and further promotes membrane lipid peroxidation. In the present study, IW maintained the stability of the cell membrane by delaying the increase of DGDG and PA and inhibiting the decrease of PC, PE, and LPC (Figure 3). The inhibitory effect of intermittent temperature treatment on PC degradation and PA accumulation was significant, indicating that this treatment can reduce the accumulation of MDA and maintain the stability of the membrane system.

Phospholipids are linked to a variety of unsaturated fatty acid chains, which together form the cell membrane. The fatty acid components of the cell membrane play a predominant role in cell signaling and defense mechanisms (Liu et al., 2011). Yao et al. (2018) found that the CI index showed significantly negative correlations with the contents of linoleic acid and linolenic acid but significantly positive correlations with stearic acid and palmitic acid during storage. The study of García-Pastor et al. (2020) showed that the MeJa treatment reduced external and internal CI symptoms in pomegranate husk likely by reducing UFA losses and enhancing the UFA/SFA ratio. It has also been reported that the GB treatment can alleviate the CI of peach fruit by increasing the levels of unsaturated fatty acids and the degree of unsaturation (Wang L. et al., 2019). During storage, cell senescence leads to lipid peroxidation, which reduces fatty acid unsaturation, resulting in decreased cell membrane fluidity and structural and functional destruction. Intermittent warming effectively maintained high relative USFA content by delaying the increase in palmitic acid content and inhibiting the decrease of linoleic acid and oleic acid content and reducing the loss of unsaturated fatty acids, thereby reducing the accumulation of membrane lipid peroxidation products, maintaining the fluidity and stability of cell membranes (Figure 4 and Table 2). Similarly, Xi et al. (2012) reported that the IW treatment could improve aroma quality by maintaining higher levels of unsaturated fatty acids in peach fruit.

The degradation of structural membrane lipids is the main cause of cell membrane loss, and PLD and LOX are two major degrading enzymes participating in this process. The activity of PLD can serve as an indicator of the intensity of hydrolysis, while LOX is a key factor in membrane lipid peroxidation (Zhang et al., 2010; Chen et al., 2011; Sirikesorn et al., 2013; Lin et al., 2016). Phosphatidic acid, released *via* catalysis by PLD, provided substrates for reactions catalyzed by LOX (Xu et al., 2016). Here, PA content in IW-treated fruit was significantly lower than that in the control, a trend also observed for the content of PLD and LOX. Intermittent warming significantly inhibited the activity of PLD and LOX as well as gene expression in the fruit, thus delaying membrane degradation (Figure 5). A similar result can be found in the research from Wang J. W. et al. (2018).

Electrolyte leakage can be used to monitor the degree of water and ion leakage due to cell rupture (Sharom et al., 1994; Marangoni et al., 1996). Malondialdehyde is a product of membrane lipid peroxidation, which is indicative of the level of membrane oxidative damage. Moreover, the content of proline is also closely related to membrane lipids (Wise and Naylor, 1987; Iba, 2002). In the present study, lower electrolyte leakage, MDA concentration, and proline concentration were observed in IW-treated fruit (Figure 2). This result showed that the IW treatment could maintain the quality of blueberry fruit by delaying membrane lipid peroxidation.

An interesting phenomenon is that we observed that some changes in control fruit were similar to the results of CI in the previous study. It was reported that the C-repeat binding transcription factor can induce the expression of genes related to hypothermia tolerance in plant cells at low temperature (Gilmour et al., 1998; Fowler and Thomashow, 2002; Ma et al., 2014). Some studies found that the *CBF* expression was positively correlated with the cold resistance of plants such as tomato and kiwifruit (Zhao et al., 2009; Ma et al., 2014). Moreover, this transcription factor participates in the control of the ABA-independent anti-cold pathway (Wong et al., 2006). We therefore determined the *VcCBF* expression level in the two groups of fruit. IW advanced the peak in *VcCBF* expression and also increased the expression levels (Figure 6), consequently improving the cold resistance of blueberry fruit while LOX activity and MDA content in treatment group were significantly lower than those in the control group. These results suggest some connection between low-temperature storage of blueberry fruit and CI. IW treatment could improve the *VcCBF* expression level and maintain membrane stability. However, this relationship needs to be further discussed in future work and there are still some deficiencies in the study. In the future, we will explore the effect of different varieties of blueberry, other treatments combined with IW on low-temperature storage of blueberry fruit, and the role of the *VcCBF* gene in this process.

## Conclusion

In conclusion, IW effectively delayed the softening of blueberry fruit and significantly delayed the incidence of pitting and decay, maintaining the storage quality of the fruit. The higher IUFA and UFA/FA content and the lower PA content in IW-treated fruit facilitated the maintenance of cellular fluidity and permeability; IW treatment also alleviated cell membrane damage by inhibiting the increased expression of *VcPLD*β and *VcLOX1*. Moreover, the gene expression level of *VcCBF* was increased in the IW-treated fruit. These results provided an experimental basis for future research as well as improved production practices for blueberry storage. In future work, we plan to study the relationship between the change in *CBF* expression and blueberry fruit quality during cold storage.

## Data Availability Statement

The original contributions presented in the study are included in the article/supplementary material, further inquiries can be directed to the corresponding author/s.

## Author Contributions

HD and YW prepared the fruit materials, participated in the physiological measurements, and participated in the statistical analyses. HD drafted the manuscript. SJ and XZ supervised the research. XK and FZ participated in the statistical analyses. QZ designed the experiment and proposed and supervised the research. All authors contributed to the article and approved the submitted version.

## Conflict of Interest

The authors declare that the research was conducted in the absence of any commercial or financial relationships that could be construed as a potential conflict of interest.
